# The immunoregulatory protein B7-H3 promotes aerobic glycolysis in oral squamous carcinoma via PI3K/Akt/mTOR pathway

**DOI:** 10.7150/jca.29838

**Published:** 2019-10-06

**Authors:** Zhangao Li, Jiyuan Liu, Lin Que, Xiufa Tang

**Affiliations:** Department of Oral and Maxillofacial Surgery, West China Hospital of Stomatology, Sichuan University, Chengdu, Sichuan, China; State Key Laboratory of Oral Diseases and National Clinical Research Center for Oral Diseases, West China Hospital of Stomatology, Sichuan University, Chengdu, Sichuan, China.

**Keywords:** OSCC, B7-H3, glycolysis, immunoregulatory protein, PI3K/Akt/mTOR pathway

## Abstract

OSCC (oral squamous carcinoma) is one of most common malignant cancer. Although previous studies have found abnormal expression of B7-H3 in human OSCC, the exact role and molecular mechanism of B7-H3 in OSCC remain unknown. In this study, we investigated the role of B7-H3 in glucose metabolic reprogramming of OSCC cells *in vitro* and *in vivo*. We first detected the expression of B7-H3 in OSCC samples. Next, siRNAs and overexpression short-hairpin RNA of B7-H3 were transfected into SCC25 and Cal27 cells, and cell proliferation, migration and invasion were analyzed via CCK8, colony formation and transwell assays. Then glycolysis flux was determined through measuring glucose uptake and lactate production, and mRNA and protein expression levels were determined by real-time quantitative PCR and western blot respectively. The results presented here showed B7-H3 was upregulated in OSCC samples compared with normal tissues, and the expression level was associated with tumor size and nodal metastasis. B7-H3 affects OSCC cell proliferation, migration and invasion. We also found that B7-H3 promoted the Warburg effect, evidenced by increase glucose uptake and lactate production. We further demonstrated that B7-H3 enhanced OSCC glycolysis through the upregulation of HIF-1α and its downstream targets, Glut1 and PFKFB3, which are key factors in glycolysis. Mechanically, we demonstrated that B7-H3 regulates HIF-1α expression through PI3K/Akt/mTOR pathway. Metabolic imaging of human OSCC cancer xenograft in mice confirmed that B7-H3 enhanced tumor glucose uptake, glycolysis promoted genes expression and tumor growth. Taken together, our results have unveiled a mechanism that B7-H3 drives OSCC progression through enhancing of glycolytic metabolic program in OSCC.

## Introduction

In 2016, 48330 new cases of head and neck cancer, one of the top ten most common cancers in the world, were estimated in America; 9570 deaths were predicted the same year 1. Oral cancer is one the most common types of head and neck cancer, 90% of which comprise the squamous carcinoma subtype 2, 3. Despite advances in surgical techniques and chemotherapies, the 5-year survival rate of oral squamous carcinoma (OSCC) is approximately 50% 2, 4. Recently, novel immunotherapeutic manipulations based on immune checkpoints have presented a promising approach to treating OSCC 5, 6. The B7 family, a class of immune checkpoint proteins primarily expressed in the plasma membrane, can interact with receptor partners in T and NK cells to mediate immune costimulatory or coinhibitory functions 7, 8. Immune checkpoint inhibitors targeting PD-1 and/or PD-L1 have been shown to be effective in clinical studies and have been regarded as second-line treatments in recurrent and metastatic OSCC 6. B7-H3 (B7 homologue 3, CD276) is a member of the B7 family, with a predominant splicing variant of 4Ig-B7-H3 in humans, which consists of exon duplication of the extracellular IgV-IgC domains followed by a transmembrane domain and a short cytoplasmic carboxyl tail with no conserved signaling motifs 7. Currently, the immunomodulatory role of B7-H3 is controversial because both costimulatory and coinhibitory functions of human 4Ig-B7-H3 on T cells have been reported in different contexts 8-10.

Studies also indicate that PD-L1 and B7-H3 promote cancer progression independent of their immunomodulatory function 10-12. Some reports have shown that B7-H3 is overexpressed in colorectal cancer, prostate cancer, breast cancer, melanoma, and osteosarcoma cells and is correlated with clinical parameters 10, 13-15. The expression level of B7-H3 can affect cancer cell proliferation, migration, and invasion as well as chemotherapy resistance 15-19. An increasing number of studies support a pro-oncogenic role for B7-H3 in various types of cancer independent of its immune function. These results clearly show that, in addition to its immunoregulatory function, B7-H3 possesses immune-independent functions that may also contribute to cancer progression.

Although how B7-H3 promotes tumors independent of immunomodulation is not completely understood, studies have shown that its downstream effectors may include NF-κB, Jak2/Stat3 and PI3K/Akt/mTOR 19, 20 . B7-H3 has been shown to regulate the secretion of the matrix metalloproteinase MMP-2 as well as TIMP-1 and TIMP-2 19, 20. B7-H3 has also been shown to increase reactive oxygen species (ROS) and HIF-1α levels to promote glycolysis in melanoma 21. Whether these tumor-promoting effects of B7-H3 are triggered by the extracellular domain remains to be determined.

B7-H3 has been shown to enhance the immune functions of T cells on OSCC cells 22, but the immune-independent functions of B7-H3 in OSCC are unclear. Other researchers have found that B7-H3 is expressed at low levels in most normal tissues but is overexpressed in OSCC and is associated with tumor progression 23, 24 These results are not in accordance with the immune costimulatory function reported in earlier studies 22 We hypothesize that B7-H3 can promote OSCC progression independent of the immunoregulatory function of T cells.

Increased aerobic glycolysis in cancer cells, known as the Warburg effect, is considered one of the most fundamental metabolic alterations during malignant transformation 25-28. It has been well accepted that dysregulated metabolic reprogramming gives cancer cell an advantage regarding proliferation and survival 29, 30. Here, we show that B7-H3 plays an important role in the regulation of cellular glucose metabolism in OSCC. Given the important roles of B7-H3 in OSCC tumorigenesis and progression, this study further demonstrates that B7-H3 may serve as an excellent therapeutic target for OSCC.

## Materials and Methods

### Patients and tissue specimens

The inclusion criteria: patients with malignant tumor who received operation in West China Hospital of Stomatology, Sichuan University and diagnosed as squamous carcinoma by pathologists. The exclusion criteria were as follows: patients who received chemotherapy, radiotherapy or any other therapy before operation, patients received biopsy before operation, patients with any other malignant tumor and recurrent patients.

OSCC tissue samples and matched adjacent healthy tissue samples from 62 patients (mean age 60.4, range 45 to 87) were obtained at the West China School of Stomatology, Sichuan University. Tumor tissues were examined by a pathologist; the tumor grade of OSCC was classified as low or high according to the 2004 WHO criteria, and the tumor stage was designated low (superficial, Ta-T1) or high (muscle invasive, T2-T4) according to the 2002 American Joint Committee on Cancer tumor node metastasis (TNM) staging system. This research was approved by the ethics board of the West China Hospital of Stomatology, Sichuan University.

### Immunohistochemistry (IHC)

Tissues were fixed with 4% paraformaldehyde (PFA), embedded in paraffin, and sliced into 5-μm thick sections, which were then deparaffinized with xylene and rehydrated in graded ethanol. For antigen retrieval, the sections were incubated in sodium citrate buffer (10 mM, pH 6.0) at 95°C for 20 min. After the sections were blocked with 5% goat serum in PBS, they were incubated overnight at 4°C with primary antibodies targeting the following proteins: B7-H3 (14453-1-AP; Proteintech; 1:100), HIF-1α (WL01607; WanLei; 1:200), for PFKFB3 (ab218121; Abcam; 1:50). For the subsequent steps, the sections were stained with SPlink Detection Kits (Biotin-Streptavidin HRP Detection Systems) (ZSGB-BIO), and the colors were developed using a DAB Kit (ZSGB-Bio) according to the manufacturer's procedures. The sections were briefly counterstained with hematoxylin, observed under a light microscope, and imaged with a digital camera. For sections that showed heterogeneous staining, the predominant pattern was taken into account for scoring. The results were analyzed simultaneously by two independent investigators. At least 4 high-power fields were chosen randomly, and 1000 cells were counted for each case. The immunohistochemical staining of these proteins in the OSCC samples was evaluated based on the ratio and intensity of the staining. The proportion score represented the estimated fraction of tumor cells positive for the stain (0 = none; 1 = less than 25%; 2 = 25-75%; 3 = greater than 75%), whereas the intensity score represented the estimated average staining intensity of positive tumor cells (0 = none; 1 = weak; 2 = moderate, 3 = intense). The overall amount of protein present was then expressed as the immunoreaction score, which was calculated by multiplying the proportion score and intensity score (ranges 0-9).

### Cell lines, culture, and transfection conditions

The cancer cell lines were purchased from ATCC. The cell lines have been tested and authenticated. SiRNAs targeting human B7-H3/HIF-1α and their negative control siRNA (siNC) were purchased from Ribobio. Transfection was performed using riboFECT™ CP (Ribobio). At 48 hours later, cells were collected for further assays. SCC25 and Cal27 cells were infected with retroviral particles containing B7-H3 or the puromycin resistance gene only (vector control) purchased from Gene. Cells were selected by 5 μM puromycin for two days to establish SCC25 and Cal27 cell lines with stable overexpression of either B7-H3 (B7-H3) or the negative control vector (NC).

### CCK8 assay

A total of 2000 cells per well were seeded in 96-well plates, siRNAs were transfected, and cell proliferation was measured using Cell Counting Kit-8 (CCK-8; Beyotime Biotechnology) according to the manufacturer's instructions. For the cells with stable overexpression, 2000 over B7-H3 or over NC cells were seeded in 96-well plates, and cell proliferation was measured. Proliferation rates were determined at 2 days and 5 days after seeding by measuring the absorbance at 450 nm with a microplate reader (Bio-Rad, USA).

### Colony formation assay

Cells (1000 per well) were seeded in 6-cm plates, siRNAs were transfected, and the cells were cultured for 14 days until large colonies were visible. During the culture period, the medium was replaced with fresh medium containing the appropriate siRNAs to ensure the silencing effect. For the cells with stable overexpression, B7-H3 overexpressing cells or its negative control cells (1000 per well) were seeded in 96-well plates and cultured for 14 days until large colonies were visible. The colonies were fixed with 4% PFA and stained with crystal violet for 5 min. The dishes were photographed, and the number of colonies formed was counted under a phase-contrast microscope.

### Transwell assay

Cell migration and invasion were measured using transwell chambers (Corning, USA) containing 24-well inserts with 8-μm pores with or without a Matrigel coating (BD Biosciences, USA) according to the manufacturer's protocol. Transfected cells (2×10^5^ per well) were seeded in the upper chamber and incubated for 24 h and 48 h for the migration and invasion assays, respectively. Then, cells in the upper chamber were removed, and the remaining cells were fixed in 4% PFA and stained with crystal violet. Cells were quantified in five randomly selected fields for each membrane, and the average cell count for three individual membranes was defined as the migration or invasion index.

### Lactate production assay

Cells (10^4^ per well) were seeded in 96-well plates and incubated overnight, and then the medium was aspirated and replaced with fresh complete medium. After 12 hours, the conditioned medium in each well was collected, centrifuged at 2500 × g at 4°C for 8 min and then assayed for lactate concentration using an L-lactate assay kit (Eton Biosciences, USA) following the manufacturer's instructions. Absolute lactate levels were calculated from the corresponding standard curve and normalized based on the cell number.

### Glucose uptake assay

Cells (10^4^ per well) were seeded in 96-well plates overnight, and the medium was aspirated and replaced with a fluorescent-tagged glucose derivative (2-NBDG) diluted in glucose-free medium. After an hour, glucose uptake was determined using a Glucose Uptake Cell-based Assay kit (Cayman Chemical, USA) following the manufacturer's instructions. The amount of 2-NBDG taken up by cells was detected by measuring the fluorescein intensity at excitation and emission wavelengths of 485 and 535 nm, respectively. Then, relative glucose uptake was calculated and normalized based on the cell number.

### Quantitative real-time PCR(qRT-PCR)

Total RNA was isolated from cultured cells using TRIzol reagent (Life Technologies). To create corresponding cDNA, DNase-treated total RNA was subjected to a RevertAid First Strand cDNA Synthesis Kit (Thermo). Quantitative real time PCR (qRT-PCR) was performed with 2*T5 Fast qPCR Mix (SYBR Green I) (TSINGKE Biological Technology, China) on a CFX96 Real-Time System with a C1000 Touch Thermal Cycler (Bio-Rad) using the following protocol: 1 min at 95°C, 40 cycles of 10 seconds at 95°C and 12 min at 60°C, and finally a 65°C to 95°C ramp up to determine the melting curve. The relative amounts of mRNA were calculated using the comparative Ct method.

### Western blot analysis

Western blotting was performed according to previously described procedures 23. Anti-B7-H3 (#14058), anti-p-mTOR (phospho-Ser2448) (#5536), and anti-HIF-1α (#36169) antibodies were purchased from Cell Signaling Technology; anti-Glut1 (21829-1-AP) antibodies from Proteintech; anti-PFKFB3 (ab218121) antibody from Abcam; anti-p-PI3K p85 (phospho-Tyr458) (orb106105) antibody from Biorbyt; anti-p-Akt(phospho-S473) (BS-4007) and anti-mTOR (BS3611) antibodies from Bioworld; and anti-PI3K(WL01169) and anti-Akt (WL0003b) antibodies from WanLei. HRP conjugated goat anti-mouse IgG and goat anti-Rabbit IgG secondary antibodies were purchased from SAB Signaling antibody.

### Xenograft tumor studies

Mouse experiments were performed in accordance with protocols approved by the Institutional Animal Care and Use Committees of University of West China Hospital of Stomatology, Sichuan University. Four-week-old female BALB/c nude mice were used in the experiments. A total of 1×10^6^ cancer cells suspended in 200 μL of PBS was injected into the right scapular region of each mouse. After the development of palpable tumors, the diameter of each tumor was measured weekly using digital micrometer calipers, and the tumor volume was calculated using the following formula: volume (mm^3^) = (W^2^ X L)/2, where W and L are the minor and major diameters, respectively.

### Glucose uptake analysis via *in vivo* fluorescent imaging

Six weeks after cell injection, the mice were intravenously injected with 10 nmoles/100 μL of XenoLight RediJect 2-DeoxyGlucosone (DG)-750 (XenoLight, PerkinElmer). At 24 hours after dye injection, the mice were analyzed for glucose uptake via fluorescent imaging using an IVIS-200 camera system (Xenogen). Ten minutes before *in vivo* imaging, mice were anesthetized with 1% pentobarbital. The imaging results were analyzed using Living Image software. A region of interest was manually selected over relevant regions of signal intensity, and the intensity was recorded as the efficiency of glucose uptake.

### Statistical analysis

All data are based on three experimental repeats unless otherwise stated. The results are expressed as the mean±SD. Statistical analysis was conducted with SPSS 17.0. Statistical significance for the experimental and control groups were determined with paired Student's t test. Comparison of the clinicopathological features and B7-H3 expression was evaluated by independent samples t test and one-way analysis of variance (ANOVA). Statistical differences of P < 0.05 (two-sided) were considered significant.

Graph Pad Prism software version 5.0 was used to create the figures.

## Results

### B7-H3 is overexpressed in OSCC and OSCC cell lines

**Figure 1:**
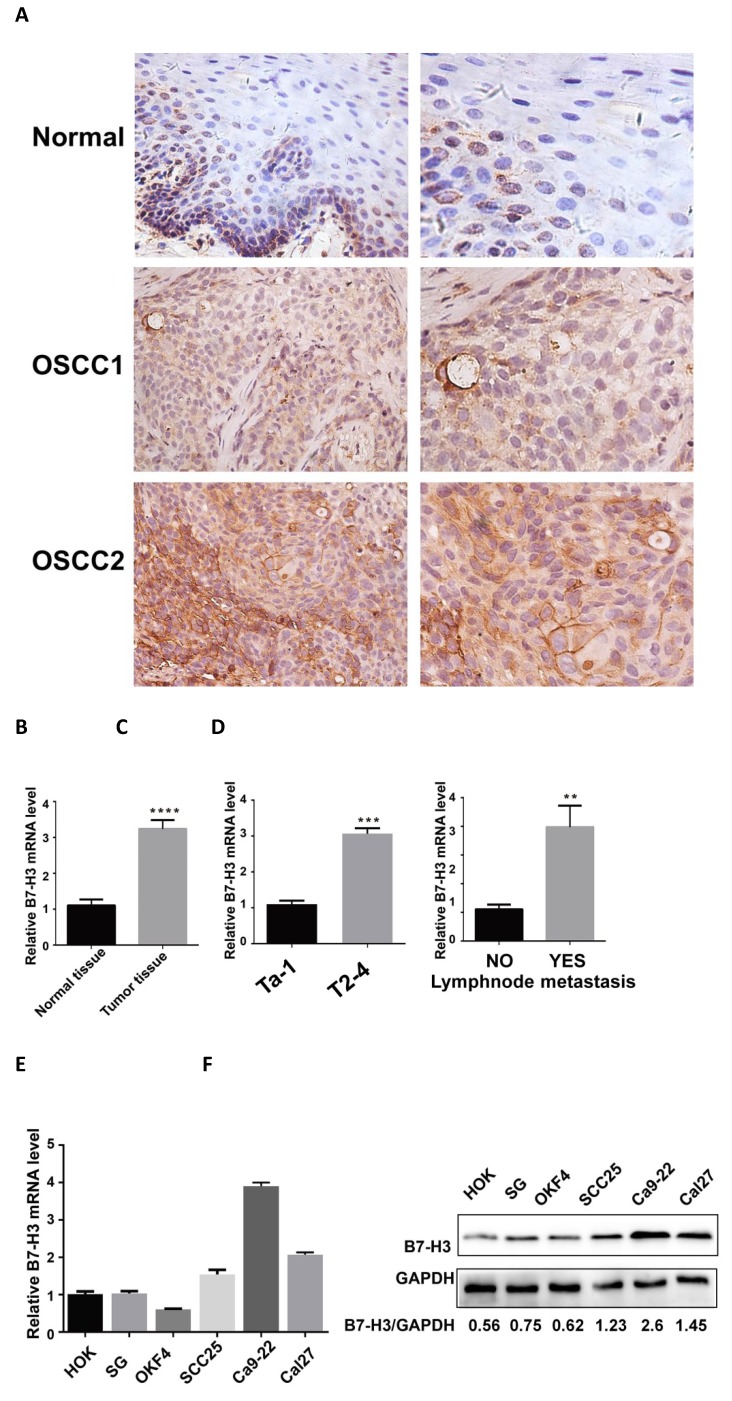
Upregulation of B7-H3 in OSCC specimens. A B7-H3 protein expression in human OSCC and adjacent healthy tissues was assessed by IHC (weakly expressed in normal tissues, moderately expressed in OSCC1, intensely expressed in OSCC2). B B7-H3 mRNA expression in human OSCC and adjacent healthy tissues was assessed by qRT-PCR. B7-H3 mRNA expression was upregulated in human OSCC than adjacent healthy tissue. C and D The association between B7-H3 mRNA expression and the clinicopathological characteristics in human OSCC was analyzed. High expression of B7-H3 mRNA in human OSCC was associated with advanced T stage and lymph node metastasis. E B7-H3 mRNA expression in OSCC cell lines was upregulated than normal epithelial cell lines. F B7-H3 protein expression in OSCC cell lines was upregulated than normal epithelial cell lines.

A previous study reported that B7-H3 is significantly overexpressed in OSCC cancer samples and was associated with increased T stage and advanced clinical stage 23. We collected 62 OSCC specimens and corresponding adjacent healthy tissues and tested B7-H3 expression via IHC and qRT-PCR. The protein level of B7-H3 was mainly located on the membrane with slight expression in the cytoplasm (Fig 1A). B7-H3 protein levels were significantly higher in tumor tissues than in the corresponding adjacent normal tissues (Table 1) and were significantly associated with increased T stage (P<0.001), lymph node metastasis (P=0.002) and recurrence (P=0.033) (Table 2). The mRNA level of B7-H3 was significantly higher in tumor tissues than in corresponding adjacent normal tissues and was significantly associated with increased T stage (P<0.001) and lymph node metastasis (P=0.003) (Figure 1B-D). No significant association was found between B7-H3 expression in OSCC and patient age, sex, cancer location, differentiation, alcohol consumption, or smoking status.

We have tested B7-H3 expression in three normal oral epithelial cell lines (HOK, SG and NOK) and three OSCC cell lines (SCC25, Ca9-22 and Cal27) from different anatomical areas through qRT-PCR and western blot (Figure 1 E and F). The mRNA and protein levels of B7-H3 were higher in OSCC cell lines than normal epithelial cell lines. Then we pick SCC25 and Cal27 for further experiment.

### B7-H3 regulates the proliferation, colony formation, migration and invasion of OSCC cells

**Figure 2:**
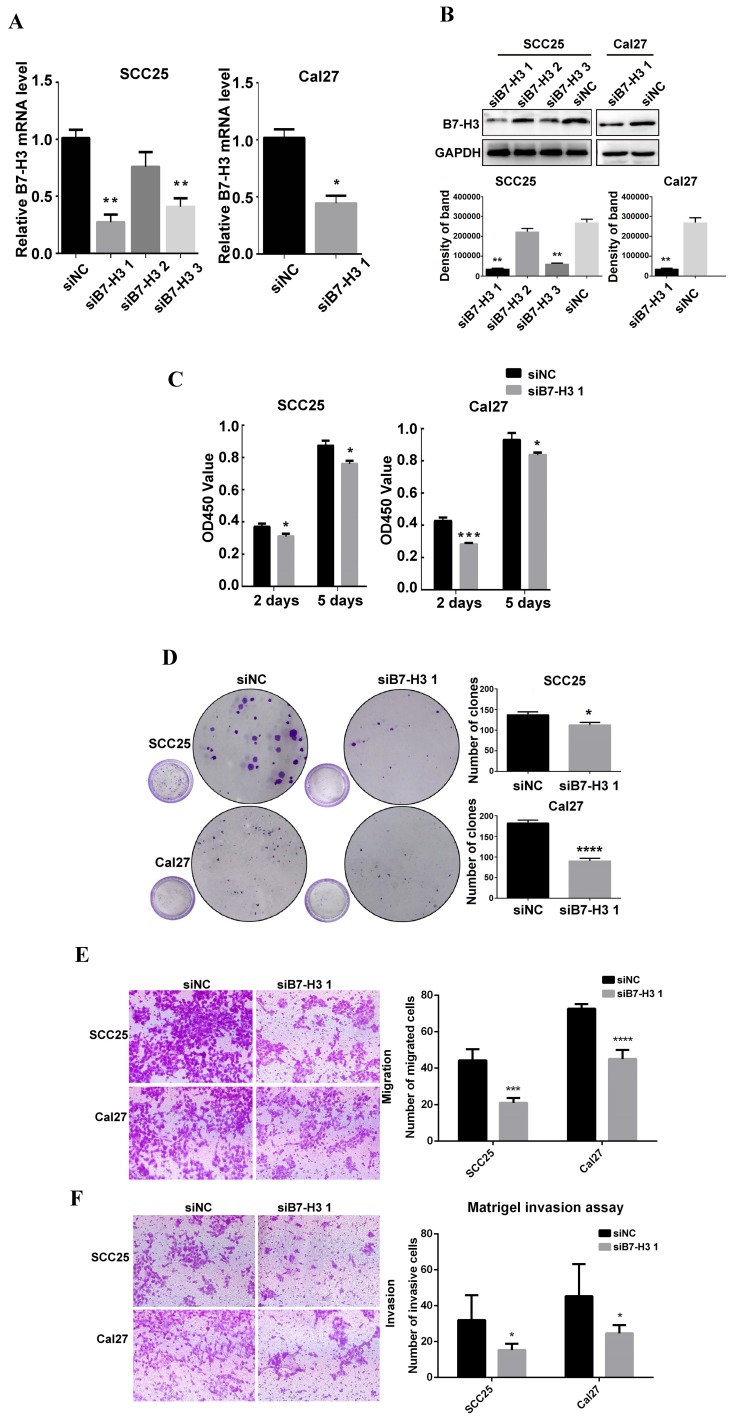
B7-H3 silencing suppressed OSCC cell proliferation, migration and invasion. A B7-H3 mRNA was evaluated by qRT-PCR 48 h after siRNA transfection. siB7-H3 1 transfection significantly decreased B7-H3 expression at the mRNA level in SCC25 and Cal27 cells. B B7-H3 protein was evaluated by western blot 72 h after siRNA treatment. SiB7-H3 1 transfection significantly decreased B7-H3 expression at the protein level in SCC25 and Cal27 cells. C The proliferation rate was observed by CCK8 assays at 2 days and 5 days after B7-H3 silencing. B7-H3 silencing remarkably slowed the proliferation rate. D Colony formation assays showed that B7-H3 silencing inhibited the colony formation of SCC25 and Cal27 cells. E The migratory ability was evaluated by transwell assays. Silencing B7-H3 reduced the migration of SCC25 and Cal27 cells. F Invasion was evaluated with Matrigel-coated transwell assays. Silencing B7-H3 reduced the invasive ability of SCC25 and Cal27 cells.

**Figure 3:**
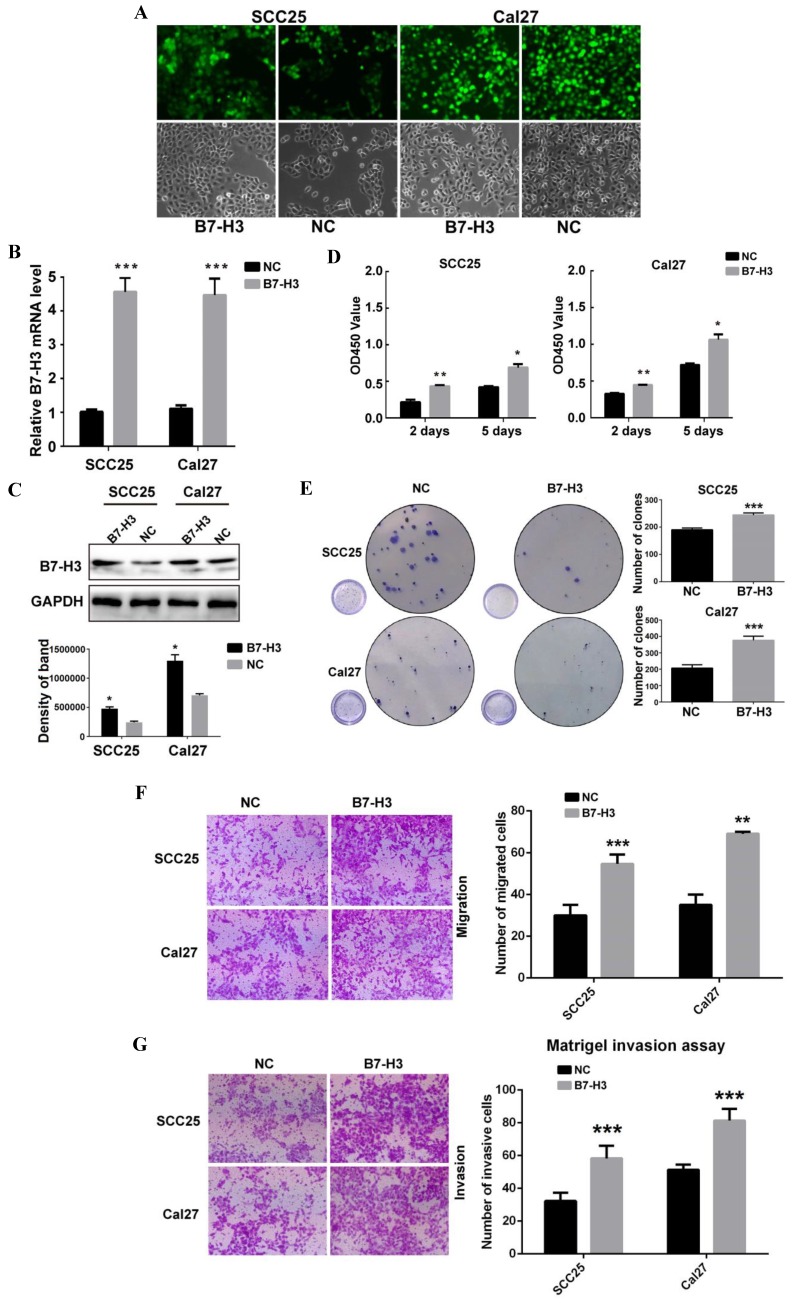
B7-H3 overexpressing promoted OSCC cell proliferation, migration and invasion. A OSCC cell lines transfected with shRNA and selected with puromycin were observed under a fluorescence microscope: the transfection rates in both cell lines were more than 90%. B, C Overexpression of B7-H3 by shRNA in SCC25 and Cal27cells was validated by qRT-PCR and western blot. D Proliferation was determined by CCK8 assays at 2 days and 5 days after seeding. B7-H3 overexpression remarkably increased the proliferation rate. E Colony formation assays showed that B7-H3 overexpression promoted colony formation in OSCC. F Migration was evaluated by transwell assays. Overexpression of B7-H3 increased the migratory ability of SCC25 and Cal27 cells. G Invasion was evaluated with Matrigel-coated transwells. Overexpression of B7-H3 increased the invasive ability of SCC25 and Cal27 cells.

To study the role of B7-H3 in OSCC cancer cells, we used siRNA to downregulate B7-H3 expression in the OSCC cell lines SCC25 and Cal27. Three siRNAs were used for SCC25 cells. The mRNA and protein expression levels of B7-H3 were analyzed after siRNA transfection to select the most effective siRNA. In SCC25 cells transfected with B7-H3-targeted siRNA, the expression level was markedly decreased 48 h after transfection compared to that in cells transfected with negative control based on the qRT-PCR data (Figure 2A). A similar decrease was found in the protein expression in cells at 72 h after B7-H3 knockdown based on the western blot results (Figure 2B). These data indicate that downregulating B7-H3 via RNA interference was specific and efficient. We selected siB7-H3 1 for subsequent assays because this siRNA exhibited the greatest silencing effect among all the siRNAs tested. Next, the silencing effect of siB7-H3 1 in Cal27 cells was confirmed by qRT-PCR and western blot (Figure 2A and B). Then, we established SCC25 and Cal27 cell lines stably overexpressing B7-H3 via short hairpin RNA (shB7-H3 and shNC), and the transfection efficiency was greater than 90% in both the SCC25 and Cal27 cell lines based on observations under a fluorescence microscope (Figure 3A). Then, the overexpression effect of shB7-H3 was confirmed by qRT-PCR and western blot (Figure 3B and C).

To characterize the role of B7-H3 in the proliferation of OSCC, CCK8 and colony formation assays were performed. SCC25 and Cal27 cells transfected with siB7-H3 1 exhibited dramatically suppressed proliferation (Figure 2C) and colony formation (Figure 2D), while cells with B7-H3 overexpression showed a significant oppositional effect (Figure 3D and E). To determine whether B7-H3 modulates tumor migration and invasion, transwell assays were performed. Silencing B7-H3 decreased the number of migratory SCC25 and Cal27 cells compared with that of the corresponding control groups (Figure 2E). Conversely, B7-H3 overexpression increased the number of migratory SCC25 and Cal27 cells in cells compared with that in the respective control groups (Figure 3F). Using a Matrigel-coated Boyden chamber assay, we found that the number of invasive cells decreased following B7-H3 silencing in SCC25 and Cal27 cells (Figure 2F). B7-H3 overexpression increased the number of invasive SCC25 and Cal27 cells (Figure 3G). Consistent with the CCK8 assay data, the cell migratory and invasion abilities of both OSCC cell lines showed decreases upon silencing of B7-H3 and increases with B7-H3 overexpression. In summary, these data demonstrated the ability of B7-H3 to enhance the proliferation, colony formation, migration and invasion of OSCC cells.

### B7-H3 promotes aerobic glycolysis in OSCC cells

**Figure 4:**
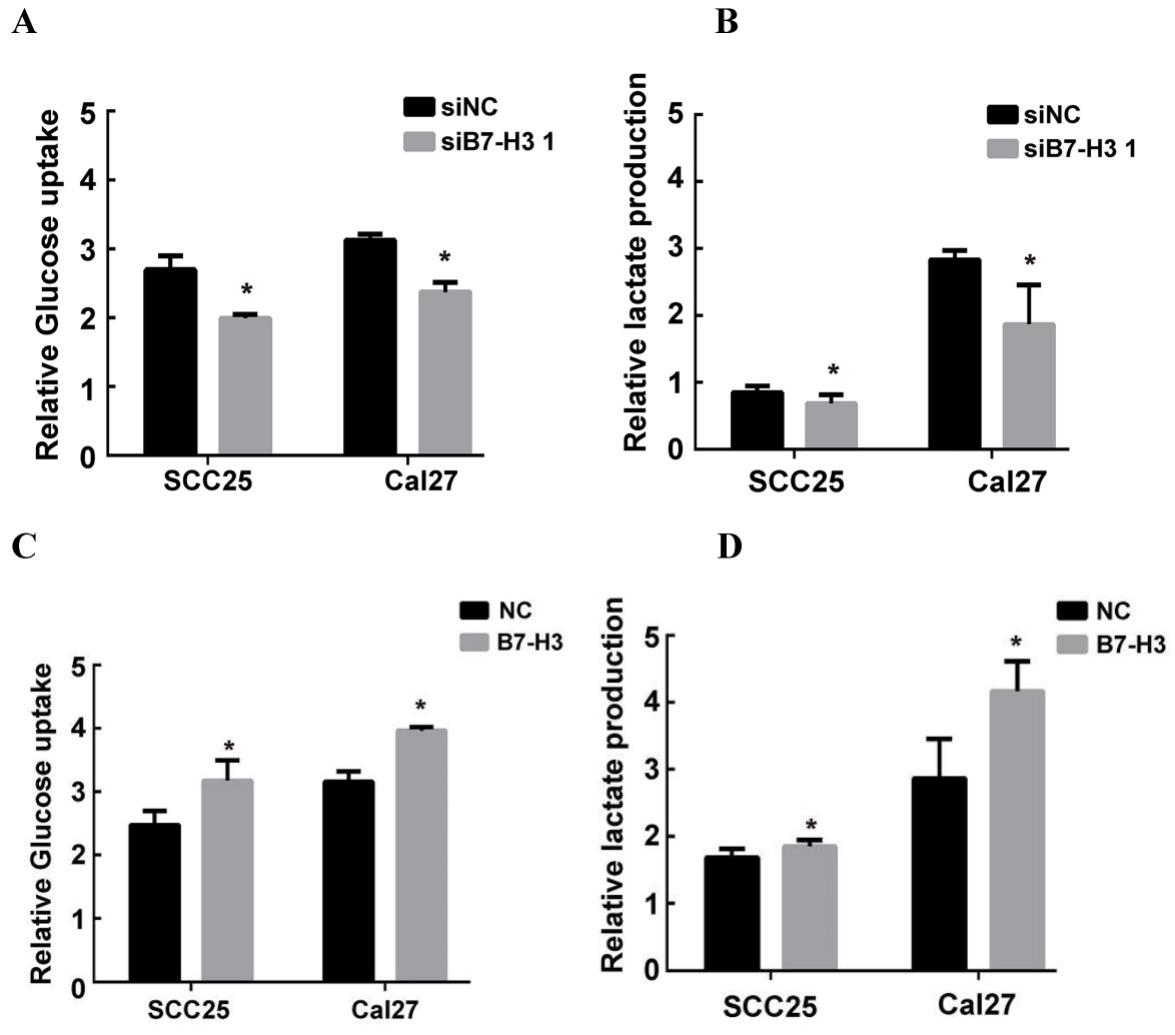
B7-H3 regulated aerobic glycolysis in OSCC cells. Glucose uptake and intracellular lactate production were detected in B7-H3-silenced cells and B7-H3-overexpressing cells. A, B B7-H3 silencing suppressed glucose uptake of and intracellular lactate production in SCC25 and Cal27 cells. C, D Overexpression of B7-H3 promotes glucose uptake and intracellular lactate production in SCC25 and Cal27.

It has been reported that B7-H3 can promote the Warburg effect in melanoma, which is an essential step in the progression of cancer 21. To study the possible mechanism by which B7-H3 regulates the proliferation, migration and invasion in OSCC, glucose uptake and intracellular lactate production were examined to measure the glycolytic activity of SCC25 and Cal27 cells with silenced or overexpressed B7-H3. Silencing B7-H3 markedly decreased intracellular lactate production and glucose uptake in both SCC25 and Cal27 cells (Figure 4A and B). In contrast, B7-H3 overexpression increased glucose uptake and intracellular lactate production (Figure 4C and D). These data indicate that B7-H3 can enhance glycolysis in OSCC.

### B7-H3 regulates glycolysis through HIF-1α

**Figure 5:**
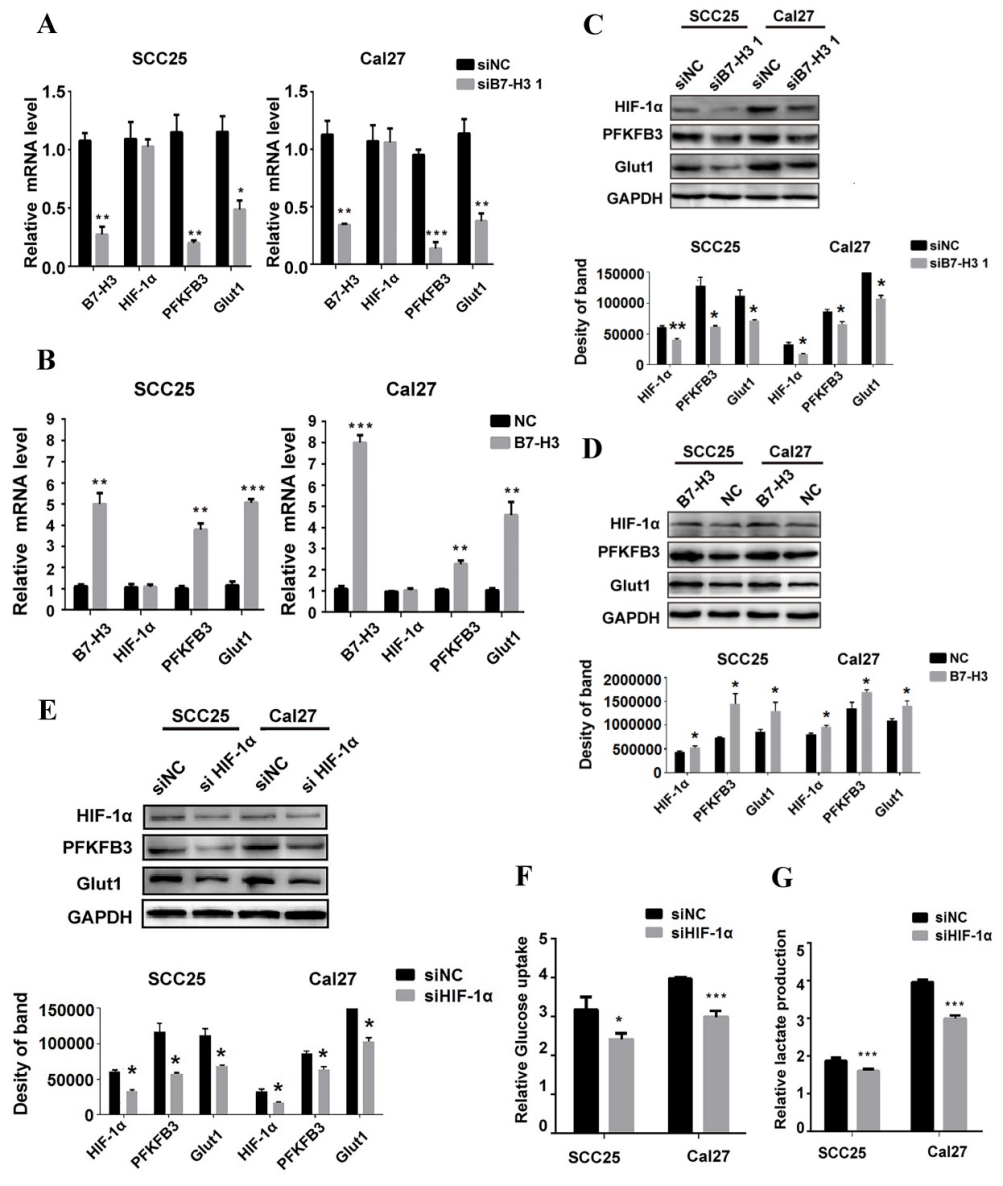
B7-H3 regulates glycolysis through HIF-1α and its downstream targets. QRT-PCR and western blot were used to determine the mRNA and protein expression of HIF-1α and key factors regulated by B7-H3 silencing and overexpression. A Silencing B7-H3 downregulated the mRNA level of PFKFB3 and Glut1 without affecting HIF-1α mRNA level. B Overexpression of B7-H3 upregulated the mRNA of PFKFB3 and Glut1 without affecting HIF-1α mRNA level. C Silencing B7-H3 downregulated the protein level of HIF-1α and its downstream targets PFKFB3 and Glut1. D Overexpression of B7-H3 upregulated the protein level of HIF-1α and its downstream targets PFKFB3 and Glut1. E Western blot analysis of HIF-1α, PFKFB3 and Glut1 expression in B7-H3-overexpressing cells when HIF-1α was silenced by siRNA. Silencing HIF-1α downregulated the expression of PFKFB3 and Glut1 in B7-H3-overexpressing cells. F, G Glucose uptake and intracellular lactate production were detected in B7-H3-overexpressing cells when HIF-1α was silenced. Silencing HIF-1α decreased glucose uptake and lactate production in B7-H3-overexpressing cells.

HIF-1α functions as a master regulator in the reprogramming of cancer metabolism in favor of aerobic glycolysis. To test whether B7-H3 regulates OSCC glycolysis through HIF-1α, qRT-PCR and western blot were performed to test the expression of HIF-1α and its downstream targets, PFKFB3 and Glut1, key factors in the glycolytic pathway. Silencing or overexpressing B7-H3 had no effect on the mRNA level of HIF-1α (Figure 5A and B); however, the HIF-1α protein level decreased in the B7-H3-silenced OSCC cells (Figure 5C), whereas it increased in the B7-H3-overexpressing OSCC cells (Figure 5D). Moreover, the expression of PFKFB3 and Glut1 at both the mRNA and protein levels decreased in the B7-H3-silenced cells (Figure 5A and C), whereas their expression increased at both the mRNA and protein levels in the B7-H3-overexpressing OSCC cells (Figure 5B and D). Then, we silenced HIF-1α in the B7-H3-overexpressing SCC25 and Cal27 cells (Figure 5E), and the glycolytic flux and gene expression were measured. The results showed that silencing of HIF-1α can decrease glucose uptake (Figure 5F) and lactate production (Figure 5G) and downregulate the expression of PFKFB3 and Glut1 (Figure 5E) in B7-H3-overexpressing cells. Altogether, these data demonstrate that B7-H3 enhances OSCC glycolysis through increasing HIF-1α protein expression.

### B7-H3 regulates aerobic glycolysis of OSCC through PI3K/Akt/mTOR signaling

**Figure 6:**
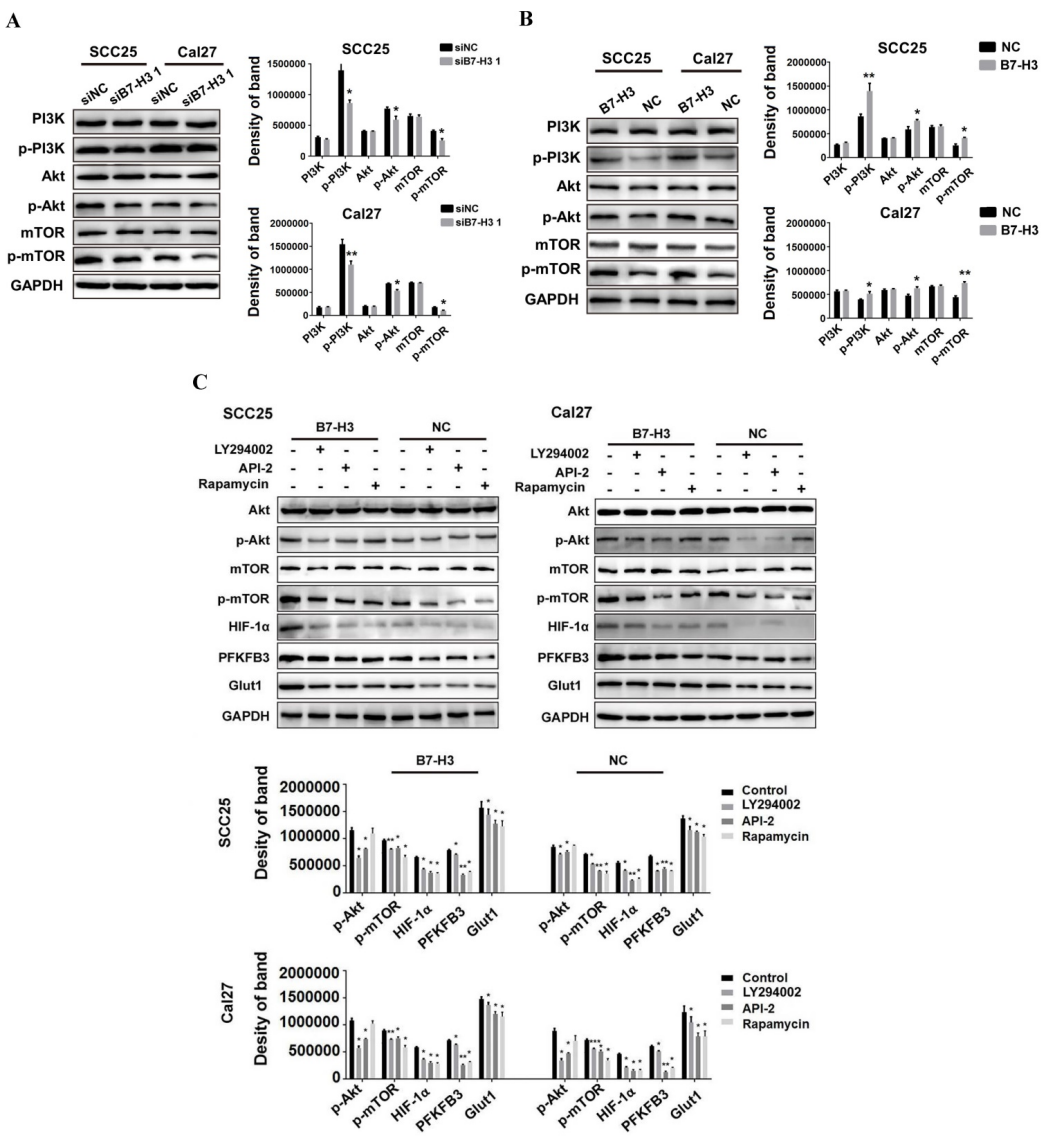
B7-H3 regulates aerobic glycolysis of OSCC through PI3K/Akt/mTOR signaling. A The protein expression of PI3K, Akt, and mTOR 72 h after siRNA treatment using siB7-H3 1 or siNC was determined via western blot. Silencing B7-H3 downregulated the expression of p-PI3K, p-Akt and p-mTOR. B The protein expression levels of PI3K, Akt, and mTOR in B7-H3-overexpressing and normal control cells were determined via western blot. Overexpression of B7-H3 upregulated the expression levels of p-PI3K, p-Akt and p-mTOR. C Protein expression of Akt, mTOR, HIF-1α and downstream glycolysis key factors in B7-H3-overexpressing cells was determined via western blot 3 h after blockade of the PI3K/Akt signaling pathway using LY294002, API-2 and rapamycin.

We next investigated how B7-H3 regulates HIF-1α protein. As previous studies have shown that B7-H3 acts upstream of the PI3K/Akt signaling pathway and that PI3K/Akt/mTOR signaling can regulate glycolysis by increasing the translation of HIF-1α, we examined the expression of PI3K, Akt and mTOR. We found that silencing B7-H3 downregulated the expression of p-PI3K, p-Akt and p-mTOR in both the SCC25 and Cal27 cells (Figure 6A) and that B7-H3 overexpression upregulated the expression of p-PI3K, p-Akt and p-mTOR (Figure 6B). Furthermore, altering B7-H3 had no effect on the expression of PI3K, Akt and mTOR (Figure 6A and B).

To explore whether inhibiting PI3K/Akt/mTOR signaling could block the upregulation of HIF-1α and its downstream targets, the PI3K/Akt/mTOR inhibitors LY294002 (PI3K inhibitor, 20 μM), API-2 (Akt inhibitor, 20 μM) and rapamycin (mTOR inhibitor, 2.5 μM) were used in B7-H3-overexpressing cells and their corresponding negative control cells. Blocking PI3K activity with LY294002 completely abolished B7-H3-promoted activation of Akt and mTOR (Figure 6C), treatment with the Akt inhibitor (API-2) blocked B7-H3-induced phosphorylation of both Akt and mTOR, and rapamycin, a specific inhibitor of mTOR, blocked B7-H3-induced mTOR activation but did not exert any obvious effects on Akt activation (Figure 6C). Additionally, blocking PI3K, Akt or mTOR also remarkably attenuated B7-H3-induced upregulation of HIF-1α, PFKFB3 and Glut1 expression (Figure 6C). Taken together, these findings suggest that B7-H3 exerts its biological effects to induce glycolytic activity in SCC25 and Cal27 cells via the PI3K/Akt/mTORC1/HIF-1α signaling axis.

### B7-H3 promotes glucose uptake and tumor growth in OSCC tumor xenografts

**Figure 7:**
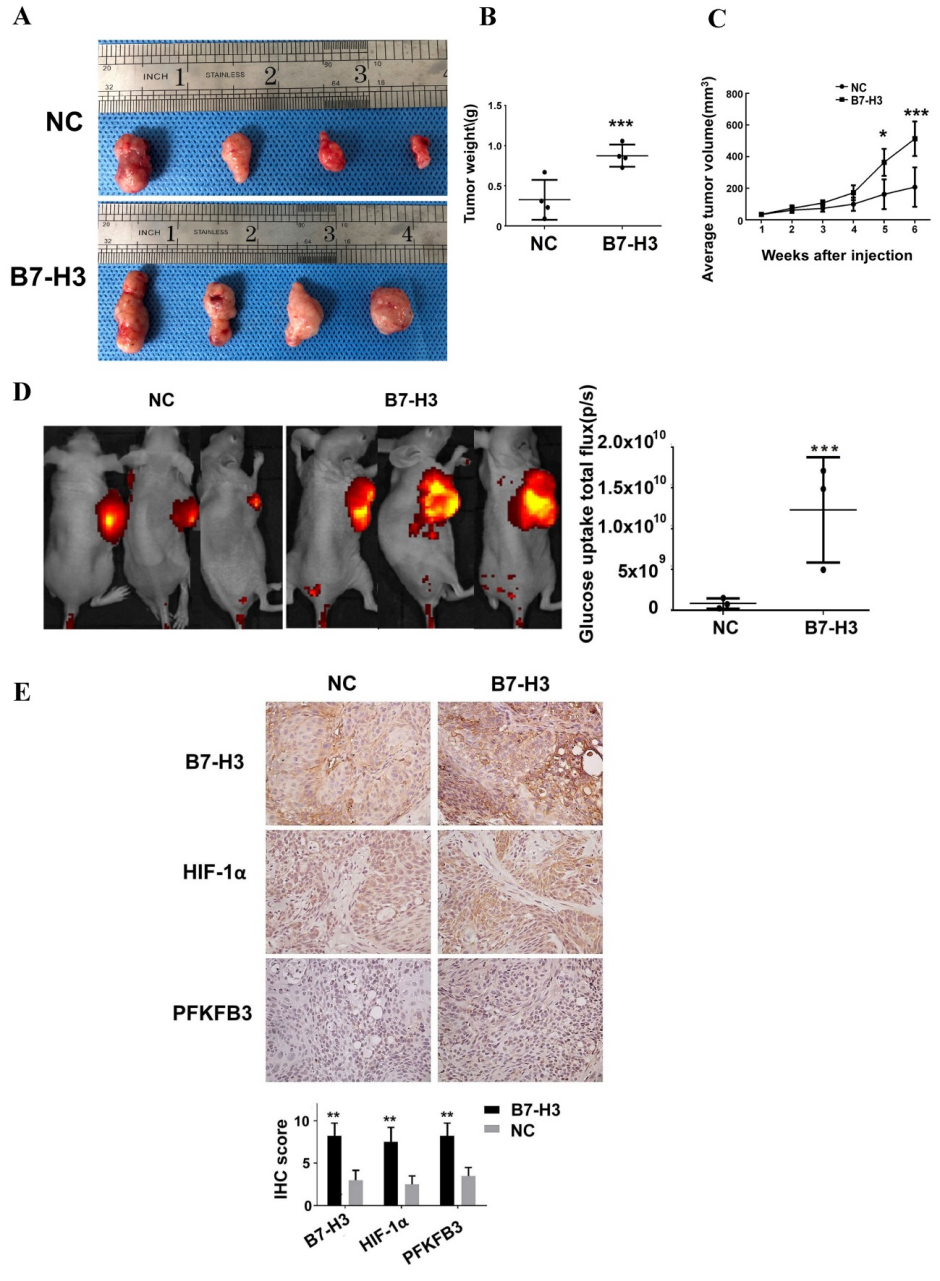
B7-H3 promotes glucose uptake and tumor growth in OSCC tumor xenografts. Cal27 B7-H3-overexpressing OSCC cells were subcutaneously implanted in athymic nude mice (n=4/group). A Images of the tumor specimens. B Tumor volume was monitored by caliper measurements for 6 weeks after injection. C Average tumor weight measured at 6 weeks after injection. Mice in the B7-H3-overexpressing group had a larger tumor weight. D Glucose uptake was measured *in vivo* at 6 weeks after tumor cell injection using the fluorescent probe 2-DG-750, as described in Materials and Methods (n=3/group; two of the mice died before fluorescent imaging). IVIS images of 2-DG-750 uptake in each individual mouse. Average of total flux (photons/second) indicates the intensity of 2-DG-750 uptake at the primary site of cell injection. Higher 2-DG-750 uptake in the B7-H3-overexpressing group was detected. E The expression levels of B7-H3, HIF-1α and PFKFB3 in implanted tumors were detected by IHC. Intense B7-H3 staining in the B7-H3-overexpressing group and weak B7-H3 staining in the control group were observed at the end point. Similarly, the expression levels of HIF-1α and PFKFB3 in the B7-H3-overexpressing group were higher than those in the control group.

To examine whether B7-H3 promotes glucose uptake and tumor growth in a mouse model, we used the B7-H3-overexpressing Cal27 OSCC cells to monitor tumor growth and glucose uptake *in vivo*. XenoLight RediJect 2-DeocyGlucosone (DG)-750, a fluorescent probe that contains four molecules of 2-deoxyglucose (2-DG) per dye molecule, was used to visualize the rate of glucose uptake by tumors. B7-H3-overexpressing Cal27 cells grew significantly larger tumors than did the control cells (Figure 7A-C). Moreover, we observed a significant increase in glucose uptake in B7-H3-overexpressing OSCC cells compared with the negative control cells (Figure 7D). Furthermore, immunohistochemical analysis of xenograft tumors confirmed that HIF-1α and PFKFB3 expression was upregulated in B7-H3-overexpressing tumors compared to that the control group (Figure 7E), which supports our *in vitro* findings showing that B7-H3 plays a critical role in enhancing aerobic glycolysis in OSCC.

## Discussion

Although most studies initially focused on the immunologic role of B7-H3 interacting with immune cells, an increasing number of studies have revealed the intrinsic pro-oncogenic role of B7-H3 independent of its immunoregulatory functions 10, 11, 17, 19-21. Here, we demonstrated that B7-H3 overexpression in OSCC was associated with T stage, lymph node metastasis and recurrence. We found that B7-H3 regulates OSCC glucose metabolism through the PI3K/Akt/mTOR pathway, which contributes to enhanced tumor cell proliferation, migration and invasion. Furthermore, in a xenograft mouse model, we showed that B7-H3 promotes OSCC tumor growth and glucose uptake *in vivo*. These findings provide insight into the role of B7-H3 in the progression of OSCC.

In this study, we found that B7-H3 is overexpressed at both the mRNA and protein levels in OSCC and is associated with T stage, lymph node metastasis and recurrence. This is in accordance with a previous study in OSCC 23, which showed B7-H3 overexpression at the protein level that was associated with increased T stage, advanced clinical stages and worse survival. Next, through B7-H3 silencing and overexpression, we found that B7-H3 can promote OSCC cell proliferation, migration and invasion, which is also consistent with a previous study showing that B7-H3 knockdown can suppress tumor xenograft growth *in vivo*. Based on these data, we believe that B7-H3 can serve as a prognostic marker and a potential therapeutic target of OSCC 31. Therefore, we further explored the mechanism by which B7-H3 promotes OSCC progression. We found that B7-H3 can promote the Warburg effect in OSCC. Studies have shown that inhibiting glycolysis can decrease the proliferation, migration and invasion of OSCC 32 and that enhancing glycolysis can promote OSCC progression by increasing its migration, invasion and stem-like cell properties 33. The role of B7-H3 in enhancing glycolysis corresponds with its function of promoting proliferation, migration and invasion in OSCC. We can conclude that B7-H3 promotes OSCC progression by enhancing the Warburg effect. Other mechanisms may also exist, which require further study.

HIF-1α plays a central role as an integrator of pathways involved in glycolysis^21^. This study has shown that the PI3K/Akt/mTOR pathway was activated by B7-H3 in OSCC. As one complex of mTOR, mTORC1 can regulate HIF-1α translation without changing its mRNA level 34. HIF-1α can promote the translation of downstream genes including Glut and key enzymes in glycolysis such as HK and PFK 21. Our results revealed that B7-H3 regulates the HIF-1α protein level as well as the mRNA and protein levels of its direct targets, PFKFB3 and Glut1. Thus, we demonstrated that B7-H3 enhances OSCC glycolysis by promoting HIF-1α translation via the PI3K/Akt/mTOR pathway; however, in a previous study, B7-H3 was shown to promote glycolysis by increasing the stability and activity of HIF-1α 21. This result may be explained by the aberrant glycosylation of B7-H3 in OSCC 23. However, lacking knowledge of any B7-H3 receptors and/or binding partners, the mechanism linking B7-H3 to PI3K/Akt/mTOR signaling and key factors remains unclear.

Our findings on the role of B7-H3 in cellular glucose metabolism reveal an immune-independent function, which can explain the cancer-promoting role of B7-H3 despite its immune costimulatory function. Currently, phase I clinical trials of the anti-B7-H3 antibody 8H9 and MGA271 in several types of cancers are undergoing testing 35-37. However, it is unknown whether the two checkpoint inhibitors can also affect the intrinsic functions of B7-H3. Our study indicates that therapeutic agents designed to inhibit the intrinsic tumor-promoting function of B7-H3 are promising for cancer treatment.

Studies have demonstrated that metabolic competition exists between cancer cells and immune cells, and the differentiation and activation of distinct subsets of immune cells are tightly modulated by specific metabolic programs 34,38-44. Based on this evidence, it is tempting to hypothesize that in the OSCC microenvironment, B7-H3 might also favor tumor growth by manipulating cancer immunosurveillance through alterations in the metabolism of cancer cells and immune cells. Studies have shown that T cell activation is accompanied by the Warburg effect, and metabolic competition in the tumor microenvironment may lead to T cell anergy, which may be a driver of cancer progression 40, 41, 44. Based on this, we suggest that suppressing B7-H3 in OSCC may alter the immunosuppressive state of the tumor by regulating cancer cell glycolysis.

Future studies should investigate whether B7-H3-induced metabolic reprogramming also plays a role in the regulation of cancer immunity to favor tumor growth and metastasis in OSCC. Although B7-H3 has been proved to be an immune costimulator in OSCC, the holistic function of B7-H3 in the OSCC needs to be clarified.

## Figures and Tables

**Table 1:** B7-H3 protein IHC reaction score in OSCC tissues and adjacent healthy tissue.

Classification	Case	B7-H3 IHC score ±SD	P
Normal tissue	62	2.23±1.38	0.000***
Tumor tissue	62	6.53±1.82	

There is a significant difference in the mean B7-H3 IHC reaction scores among OSCC and healthy tissue (P<0.001).

**Table 2:** Correlation between B7-H3 IHC reaction scores in OSCC samples and clinicopathological parameters of patients with OSCC.

Parameters		Case No.	B7-H3 IHC score ±SD	P Value
Age	>50 <=50	53. 9	6.58±1.83 6.22±1.79	0.584
Gender	Male female	35 27	6.69±1.84 6.33±1.80	0.454
Alcohol consumption	Y N	32 30	6.66±1.88 6.40±1.78	0.583
Cigarette smoking	Y N	35 27	6.51±1.76 6.56±1.93	0.930
Location	Tongue Buccal Gingiva Other sites	38 9 4 11	6.21±1.67 7.44±1.94 7.50±1.73 6.55±2.12	0.205^a^
Tumor stage	Ta-T1 T2-T4	12 50	5.00±1.04 6.90±1.78	0.000**
Lymph node metastasis	Y N	52 10	7.67±1.58 6.34±1.80	0.042*
Differentiation	low high	31 31	6.35±1.62 6.71±2.00	0.447
Recurrence	Y N	6 56	8.00±1.55 6.38±1.78	0.036*

Statistical significance of difference between two groups was analyzed by Student's test. a, statistical significance of difference between multiple groups was analyzed by one-way ANOVA test. P < 0.05 was considered to be significant. *, P<0.05;**, P<0.001; ***, P<0.001.
